# Evaluating the Knowledge of Endotracheal Cuff Pressure Monitoring Among Critical Care Providers by Palpation of Pilot Balloon and By Endotracheal Tube Cuff Manometer

**DOI:** 10.7759/cureus.5061

**Published:** 2019-07-01

**Authors:** Jawed Abubaker, Syed Zia Ullah, Shazia Ahmed, Aziz U Rehman Memon, Zohaib J Abubaker, Muhammad Imran Ansari, Musa Karim

**Affiliations:** 1 Internal Medicine, Dr. Ziauddin University and Hospital, Karachi, PAK; 2 Adult Cardiology, National Institute of Cardiovascular Diseases, Karachi, PAK; 3 Critical Care, National Institute of Cardiovascular Diseases, Karachi, PAK; 4 Medical Education, Dr. Ziauddin University and Hospital, Karachi, PAK; 5 Miscellaneous, National Institute of Cardiovascular Diseases, Karachi, PAK

**Keywords:** endotracheal tube, manometer, critical care, knowledge

## Abstract

Introduction

Mishandled endotracheal cuff pressure may either make ventilation difficult or cause damage to the airway. Therefore, the aim of this audit was to assess the knowledge about endotracheal cuff pressure monitoring with a manometer and manual palpation of pilot balloon among critical care providers.

Methods

This audit includes 150 critical care providers having experience of handling endotracheal tube (ETT) cuff at critical care area of National Institute of Cardiovascular Diseases (NICVD), Karachi from April 2017 to June 2017. Knowledge about endotracheal cuff pressure monitoring with the manometer and deleterious effects of mishandled ETT cuff was assessed using a self-reported questionnaire. Enrolled healthcare providers were asked to palpate the patient and cuff pressure was recorded and categorized.

Results

Out of 150 participants, 66 (44.0%) were doctors. Only 46 (30.67%) participants had prior knowledge about ETT cuff manometer and 110 (73.33%) had never used a manometer. Similarly only 42 (28.0%) had knowledge of hazardous effects of mishandled ETT cuff. Kappa coefficient of 0.155 with p=0.015 showed significant yet low agreement between participant prediction and the actual amount of air in cuff balloon. Agreement level was comparatively higher for staff as compared to doctors with a Kappa coefficient of 0.210 (p=0.018) vs. 0.133 (p=0.099).

Conclusion

In this study of knowledge and practice of ETT tube cuff pressure monitoring, we observed low levels of knowledge (30.67%), poor adherence to standard practice (73.33%) and were able to demonstrate poor agreement (Kappa coefficient 0.155; p=0.015) between the palpation method and cuff manometer measurements for assessing cuff pressure.

## Introduction

Endotracheal tube cuff pressure monitoring is an integral component of ICU (Intensive Care Unit) care. High cuff pressures have been reported to directly cause airway complications; epithelial necrosis (with fistula formation), tracheomalacia, laryngeal inflammation, and stenosis. Low cuff pressures may contribute to aspiration and are a direct cause of ineffective ventilation [1-4]. The 2011 guidelines from the American Society of Anesthesiologists recommend endotracheal cuff pressure monitoring by a manometer immediately after intubation and throughout the course of mechanical ventilation [5]. High volume, low pressure cuffed endotracheal tubes are currently in use and the intra-cuff pressure is almost equal to the pressure it exerts on tracheal mucosa. There is no consensus on what is an appropriate cuff pressure, cuff pressure range between 25 to 40 cm H_2_O has been suggested [6], but we know that tissue perfusion pressure is more than or equal to 25cm H_2_O and higher pressure will compromise tissue integrity. Tracheal ischemia, ulceration, inflammation, stenosis at the site of the injured tracheal wall, and granulation may develop as a result of excessive cuff pressures [7]. In practice, these complications can be avoided by maintaining optimal cuff pressure.

A cuff pressure greater than 34 cm H_2_O can result in decreased perfusion of the tracheal wall, whereas total obstruction of blood flow to the tracheal wall can occur at a compression pressure of 50 cm H_2_O. In fact, cuff pressure of 27 cm H_2_O may reduce blood flow to the cuff site by 75%. A minimal cuff pressure of 20 cm H_2_O has been recommended for positive-pressure ventilation and prevention of aspiration [7]. Different studies have shown the complication related to under-inflation and over-inflation of endotracheal cuff pressures. A prospective study assessing the risk factors for tracheal stenosis in patients subjected to prolonged tracheal intubation (more than eight hours in duration) concluded that strict monitoring of the cuff pressure thrice a day might help to prevent ischemic lesions and tracheal stenosis [8].

Routine monitoring of cuff pressures by manometry is now standard practice in most intensive care unit (ICU) and operating rooms (OR). Unfortunately, in Pakistan, this is infrequent. Part of the problem is a lack of awareness about the complications of cuff over or under inflation. Other factors are deficient knowledge regarding the limits of cuff pressure and monitoring methods [9]. In most occasions, endotracheal tube cuff pressures are ‘guesstimated’ by palpating the pilot balloon instead of directly measured. The objective of this study was to audit knowledge of critical care providers about manometric endotracheal cuff pressure monitoring and to compare manometric monitoring with the palpation method.

## Materials and methods

With the approval of the Institutional Ethical Review Committee, this audit was conducted in critical care areas, coronary care unit (CCU), intensive care unit (ICU), and emergency room (ER), of National Institute of Cardiovascular Diseases (NICVD), Karachi from April 2017 to June 2017. Critical care providers with at least six months experience of handling endotracheal tube (ETT) cuffs were enrolled for the study. Verbal consent for participation was obtained. On a predefined structured questionnaire, data regarding participant demographics, education, and professional experience was collected. Knowledge about manometric endotracheal cuff pressure monitoring and the deleterious effects of mishandled ETT cuff were assessed using a self-reported questionnaire. In intubated patients with already inserted ET tubes (internal diameters of 7.0 mm, 7.5 mm, and 8.0 mm), cuff pressures were measured using a Portex manometer (Smith Medical International, Minnesota, US) and then ‘estimated’ by the palpation of the pilot balloon. The cuff pressure was recorded and categorized as under-inflated (less than 20 cm H_2_O), over-inflated (higher than 30 cm H_2_O) or within the range (20 to 30 cm H_2_O). If the ETT cuff pressures were out of range, it was optimized by cuff manometer. To minimize bias, healthcare professionals not directly involved in that patient’s care were asked to assess cuff pressures.

IBM SPSS Statistics for Windows, Version 21.0 (IBM Corp., Armonk, NY, US) and SAS (version 8.1, SAS Institute, Inc., Cary, US) was used to analyze the data. Frequency and percentages were calculated for categorical variables. Chi-square test was applied to test for associations between categorical variables. Kappa coefficient was calculated to assess the agreement between the predicted and actual amount of air in the cuff balloon. Two-sided p-value ≤ 0.05 was taken as criteria for statistical significance.

## Results

Out of 150 participants, 66 (44%) were doctors, 71 (47.33%) of the participants had more than five years of experience. Only 46 (30.67%) of the participants had prior knowledge about ETT cuff manometry and 110 (73.33%) had never used a manometer. Similarly, only 42 (28%) of the participants had knowledge of the hazardous effects of mishandled ETT cuff pressures. Baseline characteristics of the study participants are summarized in Table 1.

**Table 1:** Baseline characteristics and knowledge assessment ETT = endotracheal tube

Characteristics	Frequency (%)
Designation	
Doctors	66 (44%)
Staff	84 (56%)
Shift of health care personnel	
Morning	103 (68.67%)
Evening	40 (26.67%)
Night	7 (4.67%)
Year of experience	
1-2 years	22 (14.67%)
>2-5 years	57 (38%)
> 5 years	71 (47.33%)
Knowledge about ETT cuff manometer	
Yes	46 (30.67%)
No	104 (69.33%)
Use of manometer	
More often	24 (16%)
Less often	10 (6.67%)
Rarely	6 (4%)
Never	110 (73.33%)
Knowledge of hazardous effects of mishandled ETT cuff	
Yes	42 (28%)
No	108 (72%)
Participants knowledge of air required to fill cuff balloon in mmHg	
0-2	3 (2%)
2-4	26 (17.33%)
5-8	47 ´(31.33%)
8-10	28 (18.67%)
10-15	10 (6.67%)
>15	1 (0.67%)
Not known	35 (23.33%)

**Figure 1:**
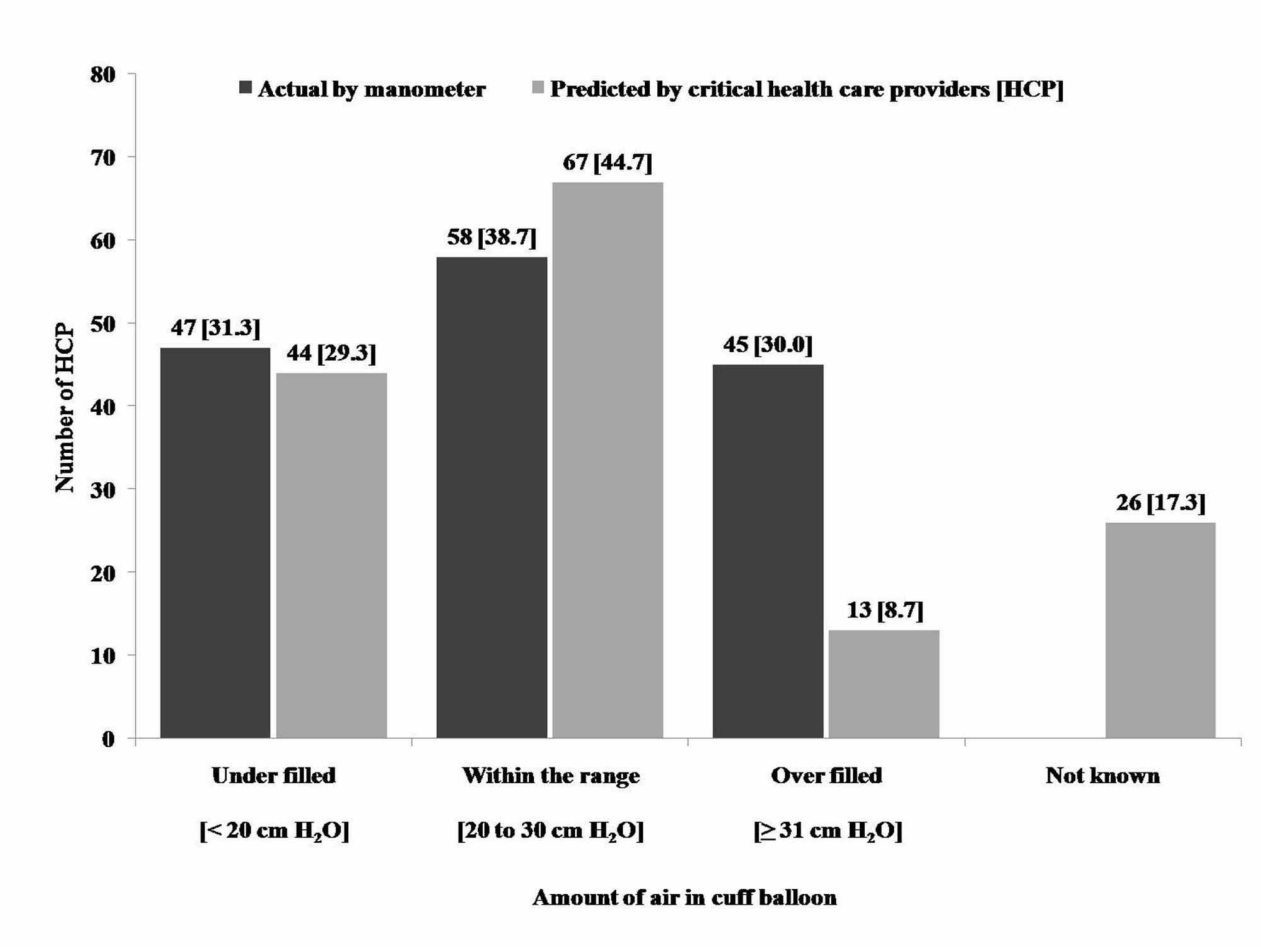
Critical care provider’s knowledge assessment of the amount of air in cuff balloon using endotracheal tube cuff manometer

Almost half of the participating critical care providers, 68 (45.33%), never did routine palpation of the ET cuff. Only 67 (44.67%) participants correctly estimated the amount of air in the cuff balloon as within the range, whilst, manometric assessment of air was within range in 58 (38.67%) cases. Knowledge assessment of the amount of air in cuff balloon of the study participants is summarized in Figure 1.

Overall Kappa coefficient was found to be 0.155 with p-value <0.05, representing a statistically significant but low agreement between the actual and estimated amount of air in cuff balloon. Nurses’ prediction of air in ET cuff balloon showed relatively more agreement than that of doctors’, 0.210 vs. 0.133. Kappa coefficient and 95% confidence integral for agreement between predicted and actual air in the cuff balloon is presented in Table 2.

**Table 2:** Kappa coefficient between predicted and the actual air in cuff balloon * Statistically significant at 5% level of significance

	Total (n=150)	Designation	
		Doctors (n=66)	Staff (n=84)
Kappa Coefficient	0.155	0.133	0.210
95% Lower Conf Limit	0.022	-0.042	0.043
95% Upper Conf Limit	0.288	0.309	0.389
p-value	0.015*	0.099	0.018*

## Discussion

Our study shows a surprisingly low level of knowledge amongst critical care providers regarding endotracheal tube (ETT) cuff pressure measurement by manometry; 46 (30.67%). A majority, 73.33% (110), had never used a manometer and very few, 42 (28%), were aware of the potentially harmful effects of inappropriate (either high or low) ETT cuff pressure. Despite various innovations and developments in recent years maintaining ETT cuff pressure at its optimal level remains one of the major challenges in the critical care setting [10]. ETT cuff pressure between 20 to 30 cm H_2_O is the recommended safe range [11], over-filling or under-filling of ETT cuff may result in various injuries and complications [12-14]. Over-inflation of ETT cuff is reported to be associated with alterations in swallowing, tracheoesophageal fistulae, stenosis and necrosis, tracheal wall ischemia, tracheal rupture, and recurrent laryngeal nerve palsy. It is also reported to be more commonly associated with a sore throat and stridor in patients after extubation [11-13]. Similarly, ventilator-associated pneumonia is associated with under-inflation of ETT cuff due to aspiration of pharyngeal secretions [12,14].

Owing to the fact that mishandling of ETT cuff pressure and under or over inflation may result in various complications in patients after intubation, which may result in increased hospitalization and healthcare burden in these patients. A number of pressure monitoring techniques employed in clinical practice are described in the literature which includes cuff pressure measurement, minimal occlusive volume, and minimal leak technique [13,14]. Cuff pressure measurement using aneroid manometer was found to be associated with lesser complications in patients after intubation [15]. Unfortunately, a significant number of critical healthcare professionals, 45.33% (68), in our sample never did the routine palpation of cuff and majority of them 73.33% (110), have never used a manometer.

Educating critical health care providers regarding the importance of monitoring cuff pressures and use of manometer is mandatory if we are to improve the delivery of our healthcare services and to reduce iatrogenic morbidity and mortality in critical illness.

## Conclusions

In this study of knowledge and practice of ETT tube cuff pressure monitoring, we observed low levels of knowledge, poor adherence to standard practice and were able to demonstrate poor agreement between the palpation method and manometric method for assessing cuff pressure. We recommend every institution should evaluate their own strategy and develop protocols to ensure optimal delivery of safe healthcare in the ICU.
